# Supplementary material for: Learning curve and surgical outcome of robotic assisted colorectal surgery with ERAS program
Source: Sci Rep. 2022 Nov 29;12:20566. doi: 10.1038/s41598-022-24665-w (PMC9709162; doi:10.1038/s41598-022-24665-w)
Supplement: Supplementary file 1 — Supplementary Information 1. [file 41598_2022_24665_MOESM1_ESM.zip › LSC Raw data-Ñ[▒K/082/H_ñw╝╨░O▒Kñσ.pdf]

正式摘要(電子簽章：000000194819774)

W95 / W95 ☐ 英文標題(注意:無英文姓名-請補病人基本檔!) ☐【讀取】憑證

臺中榮民總醫院
出院病歷摘要

轉入號 : 轉出號 :

[redacted]

(8)流水編號	
(9)入院日期	109/07/01 (2020) 直腸外科 W95 - 29
(10)轉科日期	
(11)出院日期	109/07/16 (2020) 住院天數計 15 日

診斷

- 入院 :A-COLON ADENOMA, FOR LAPA-OP
  (6/25 Lin Shin Colonoscopy: Advanced colon cancer, about 3.5cm, proximal A-colon, s/p biopsy)
- 出院 :A-colon tumor, s/p laparoscopic right hemicolectomy on 20200703, cT4aN1M0 (Pathology: Adenocarcinoma, moderately differentiated, of ascending colon. Tumor invaded to muscularis propria and metastasized to mesocolic lymph nodes (2/13). The appendix, ileocecal valve, and bilateral cut ends are free of tumor)

主訴

- general malaise, abdominal fullness and poor appetite for about 2 weeks

病史

- The patient is a 69 year old male who had past history of
  1. type 2 DM with regular medication on our FMP OPD
  2. HTN with regular medication on our FMP OPD
  3. Sinusitis s/p op about 30+ years ago

  According to the patient, he complained of general malaise, abdominal fullness and poor appetite for about 2 weeks. No bloody or tarry stool noted. Nor changes in bowel habits was told. So he came to 林新hospital first on last Thursday(6/25), where lab data showed anemia s/p blood transfusion and EGD/Colonoscopy revealed tumor over ascending colon. Surgery was suggested by the doctor. So he came to our OPD for help on 6/30. This time, he was admitted for scheduled operation of Laparoscopic right colectomy and anastomosis on 7/3.

體檢發現

-
  - General appearance :
    - acute ill looking , conscious clear , without cardiopulmonary distress
    - body weight : 64 kg , height : 157 cm
    - Vital signs自動帶入date 體溫(BT) 脈搏(P) 呼吸(R) 血壓(BP) 身高(BH) 體重(BW) BMI
      20200701 35.8℃ 79次/分 17次/分 162/77mmHg 157cm 64kg 26.0
    - Vital signs: B.P. 162/77 mmHg , P.R. 79 /min , R.R. 17 /min , B.T. 35.8 ℃
  - HEENT :
    - Eyes:
      - Conjunctiva : pale: bilateral eyes
      - Sclera : normal
    - ENT: no otorrhea, no rhinorrhea, no erythematous mucosa, no injected tonsil, no deformity
  - Neck: supple, freely movable, trachea midline,no deformity, no engorged jugular vein , no palpable thyroid or lymph node enlargement
  - Chest:
    - Inspection: Grossly normal, symmetric and fully expansion, no accessory muscle used, no operation scar, no spider angiomata, no gynecomastia
    - Palpation: no palpable mass or nodule, no subcutaneous emphysema, no tenderness
    - Ausculation: clear breathing sound

- Heart:
  - Palpation: no thrill, no heave
  - Percussion: normal heart size
  - Ausculation: regular heart beat, normal S1, S2, no gallop, no murmur
- Extremities: No deformity, full ranged of motion without limitation, no clubbing finger or nail cyanosis, no pitting edema, no ulcer or pigmentation, no obvious skin lesion, no varicose vein, no bone, joint or muscle tenderness

CRS CONDITION

- Karnofsky performance status: (90) Able to carry on normal activity; minor signs or symptoms of disease
- PE of Abdomen:
- __1. Inspection: ovoid in shape, abdominal fullness
- __2. Ausculation: normoactive bowel sound
- __3. Palapation: mild tenderness, no rebounding pain
- __4. Percussion: tympanic percussion sound
- CEA: N/A ng/ml
- Imaging Studies:
- Abnormal Lab data: Hb: 10.2
- Colonoscope report: (林新hospital)suspect A-colon tumor
- Figures:
  1. colon & rectum

手術日期及方法

- 手術日期及方法20200703 1. Laparoscopic right hemicolectomy with side-to-side anastomosis, antiperstalsis, endo-GIA 60/3.8*2, re-inforcement with silk (+).
  2. Vessel ligation : ileo-colic, right colic, and right branch of middle colic vessels.
  3. Cut margin: proximal (ileum) 8 cm, (colon) 7 cm, distal 20 cm.
  4. Repair of mesentery defect: nil.
  5. A 12Fr. penrose drain placed at morrison's pounch.

住院治療經過

- After admission, we performed operation of laparoscopic right hemicolectomy on 20200703 smoothly. We kept following up his clinical condition after the surgery, with normal penrose drainage amount and color noted. Urine output at least 1000cc per day. Though much flatus passage since POD 1, he still complained of abdominal fullness and severe cramping pain. No stool passage yet till POD 7. Follow-up KUB showed much bowel gas accumulation, compatible with his clinical condition. Poor eating and drinking status due to above symptoms. We arranged abd. sono for further evaluation and was done on 7/10, which only revealed Fatty liver, Sludge of gall bladder and multiple renal cysts about 9-11 mm in size over bil. kidneys. Otherwise, no specific finding noted. On POD8(7/11), normal stool passage for 3 times finally noted with greatly improved abdominal fullness and cramping pain. Also, due to poor intake and nutrition status, we gave him PPN use for 3 days since 7/11. Penrose drain was removed on 7/13. Final pathology report showed adenocarcinoma, moderately differentiated, of ascending colon. Tumor invaded to muscularis propria and metastasized to mesocolic lymph nodes (2/13). The appendix, ileocecal valve, and bilateral cut ends are free of tumor. Further chemotherapy explained to the patient and his family. With improving clinical condition and eating status, the patient is allowed to be discharged tomorrow under relatively stable condition.

合併症

- nil

一般檢查

- 累積報告 :

DATE	TIME	ALB	TP	BIL.T	BIL.D	ALKP	AST	ALT	LDH	CHOL	TG	HDL-C	LDL	UA	NH3	AMY
1090703	1134	3.8	7.0								117		62	5.3		
1090709	0849	3.0					39	20								
1090713	0907	3.0				84	17	18								

DATE	TIME	WBC	RBC	HGB	HCT	MCV	PLT	BLST	NEUT	BAND	SEG	LYM	MON	EOS	BAS
1090706	0917	8870	3.98	8.4	28.2	70.9	220				85.5	7.3	6.0	1.2	0.0
1090709	0846	7690		9.0			431		80.8			7.3	9.6	2.2	0.1
1090713	0850	8950	4.64	9.3	31.4	67.7	569		80.4			10.2	5.6	3.5	0.3

DATE	TIME	NA	K	CL	CA	BUN	CREAT	Mg	P	CRP	ESR	PCT	LIPASE	LACTATE
1090706	0958	132	3.7											
1090709	0849	137	3.7		7.8	8	0.97							
1090713	0907	133	4.0		7.7	15	0.98							

DATE	TIME	GLUCOSE	PAB	TRF	ZN	CU	KETONE	CK	CKMB	PT-P	PT-C	APTT-P	APTT-C	TRO-T
1090706	0922	111												

- 放射線報告 :**檢驗項目 :SONO,ABDOMEN 報告日期 :1090710**

  主　訴：
  Result:
  Finding:
  Sonography of the abdomen was performed and showed:
  1. Fatty liver
  2. Sludge of gall bladder
  3. Poor demonstration of pancreas due to bowel gas
  4. Multiple renal cysts about 9-11 mm in size over
     bil. kidneys
  5. Bil. pleural effusion
  6. No significant abnormal findings of the spleen
  7. Otherwise, no significant abnormal findings
  Impression:
  As above description.

  檢驗項目 :ABDOMEN (KUB),SUPINE 報告日期 :1090712
  主　訴：
  Result:
  Finding:
  > Some fecal material retention within the colon
  > Gaseous distention of bowel loops with fecal material retention, ileus is considered.
  > DJD change of T-L spines with marginal spurs formation
  > Surgical clips and autosuture over right upper abdomen
  > S/P drainage tube over right abdomen
  > Obscuration of bilateral psoas shadows
  Impression:
  As above description.

  檢驗項目 :ABDOMEN (KUB),SUPINE 報告日期 :1090708
  主　訴：
  Result:
  Finding:
  > Distention of GI tract is noted, ileus is considered.
  > Degenerative change of thoracic and lumbar spine with osteophytes formation
  > Surgical clips and stitches retention over right abdomen
  > S/p penrose drainage tube over right abdomen
  Impression:
  As above description.

特殊檢查

病理報告

- 活體組織切片病理報告　手術日期：**109/07/03**　病理號：**S0919338**

診斷醫師:陳志榮 報告日期:109/07/13
Pathologic diagnosis:
Colon, terminal ileum, and appendix, laparoscopic right hemicolectomy --- Adenocarcinoma, moderately differentiated, of ascending colon. Tumor invaded to muscularis propria and metastasized to mesocolic lymph nodes (2/13). The appendix, ileocecal valve, and bilateral cut ends are free of tumor.
Ancillary study for diagnosis:
IHC stains for PMS2 and MSH6 are done on section F. Both markers are preserved.
Prognostic and predictive factor:
1. Tumor size: 3.6x3.2 cm.
2. Depth of invasion: Muscularis propria.
3. Histologic grade: G2: Moderately differentiated.
4. Angiolymphatic invasion: Present.
5. Perineural invasion: Not identified.
6. Tumor deposits (discontinuous extramural extension): Not identified.
7. Macroscopic tumor perforation: Not identified.
8. Surgical Margins:

- a) Proximal Margin: Uninvolved by invasive carcinoma, distance of tumor from margin: 8.5 cm.
  b) Distal Margin: Uninvolved by invasive carcinoma, distance of tumor from margin: 11 cm.
  c) Radial or Mesenteric Margin: Uninvolved by invasive carcinoma, distance of tumor from margin: more than 1 cm.
  9. Lymph node status: Involved (2/13).
  10. Extranodal involvement: Not identified.
  11. Tumor regression grading S/P CCRT: No known presurgical therapy.
  12. Type of polyp in which invasive carcinoma arose: Not identified.
  13. Additional pathologic findings: Not identified.
  14. Pathological TNM Stage: pT2N1b(According to the eighth edition, American Joint Committee on Cancer Staging Guidelines for Tumors).
  15. TNM descriptors: Not applicable.
  Gross description:
  The specimen consists of a segment of colon, 15 cm in length, and 3 cm in proximal circumference, 3.5 cm in distal cut end circumference, and a segment of terminal ileum, 8 cm in length, and 2.5 cm in cut end circumference. An unremarkable appendix, 7x0.5x0.5 cm, is noted. A gray tan firm annular elevated and centrally ulcerative tumor, 3.6x3.2 cm, is noted, 8.5 cm to the nearest cut end. On cross section, the tumor invaded to the muscular layer with more than 10 mm to the radial margin grossly. The appendix, ileocecal valve, and both cut ends are free of tumor. Representative part for section: A) distal cut end B) proximal cut end C) ileocecal valve D) appendix E) CFM F-H) tumor and serosa J) tumor and normal colon tissue K-U) lymph nodes.
  Note:
  1. The interpretation of MMR panel and recommended studies are as below:
  (1) No loss of nuclear expression of MMR proteins: low probability of microsatellite instability-high (MSI-H) .
  (2) Please note: There are exceptions to the above IHC interpretations. These results should not be considered in isolation, and clinical correlation with genetic counseling is recommended to assess the need for germline testing.
  2. This case has been peer reviewed by two doctors.
  #T-59420_2 #M-81403_2 2 1105 M0000F
  #T-C4560_2 #M-81406_2 2 1105 000000
  #T-59200_2 #M-09183_2 0 1105 000000

其它

出院時情況

- 3.改門診治療

出院指示

藥名	劑量	劑量單位	用法	頻次	數量	數量單位	現況	天數	單位
Gaslan tab 40mg	2	TAB	PO	QID	56	TAB	TH	7	天
Mag. Oxide tab	2	TAB	PO	TID	42	TAB	TH	7	天
$Biofermin tab	2	TAB	PO	TID	42	TAB	TH	7	天
Ultracet tab	1	TAB	PO	Q6HPRN	12	TAB	TH	3	天

出院計劃

- 門診日期
  - date on 2020/07/22
- 門診追蹤

主治醫師 2062F 蔣鋒帆 / 住院醫師 9889F 陳俊男

正式摘要(**電子簽章：000000194819774)**

W95 / W95

doctorRank:B
